# Understanding Uncontrolled Eating after Bariatric Surgery: The Role of Excessive Skin and Body Image Shame

**DOI:** 10.3390/jcm10132967

**Published:** 2021-07-01

**Authors:** Marta de Lourdes, Luísa Cerqueira, Ana Pinto-Bastos, João Marôco, Lara Palmeira, Isabel Brandão, Ana Rita Vaz, Eva Conceição

**Affiliations:** 1 School of Psychology, University of Minho, 4710-057 Braga, Portugal; martamagalhaeslourdes@outlook.com (M.d.L.); luisacerqueira95@gmail.com (L.C.); anapintobastos@hotmail.com (A.P.-B.); anavaz@psi.uminho.pt (A.R.V.); 2 William James Centre for Research, ISPA–Instituto Universitário, 1100-304 Lisboa, Portugal; jpmaroco@ispa.pt; 3 Center for Research in Neuropsychology and Cognitive Behavioral Intervention (CINEICC), Faculty of Psychology and Educational Sciences, University of Coimbra, 3001-802 Coimbra, Portugal; larapalmeira@gmail.com; 4 Faculty of Medicine, University of Porto, 4200-319 Porto, Portugal; isabelmbrandao@gmail.com; 5 University Hospital Center of São João, 4200-319 Porto, Portugal

**Keywords:** bariatric surgery, excess skin, body image shame, eating-related psychopathology, uncontrolled eating, negative urgency

## Abstract

Excess skin and disordered eating behaviors are referred to as some of the major negative consequences of bariatric surgery as well as body image shame. This study sought to explore how discomfort with excessive skin, body image shame, psychological distress, eating-related psychopathology, and negative urgency interact to understand uncontrolled eating among woman submitted to bariatric surgery. A cross-sectional sample of 137 women was evaluated postoperatively through self-report questionnaires assessing discomfort with excess skin, body image shame, eating-related psychopathology, negative urgency, and uncontrolled eating in a hospital center in the north of Portugal. Pearson correlations and Structural Equation Modeling (SEM) were performed. Body image shame mediated the relationship between discomfort with excess skin and eating-related psychopathology. In turn, the relationship between eating-related psychopathology and uncontrolled eating was mediated by negative urgency. This study highlights the impact of excess skin and body image shame on eating behavior post-bariatric-surgery. Considering the proven impact of uncontrolled eating on weight-loss results post-surgery, understanding the mechanisms underlying this problem is highly important. Our findings provide helpful insight for multidisciplinary teams committed to providing care to bariatric patients struggling with body image and eating difficulties.

## 1. Introduction

Bariatric surgery is currently the most effective and durable treatment for severe obesity, resulting in significant weight loss and substantial improvements in obesity-related comorbidities [1] and patients’ psychosocial function [2]. With a variability of weight loss outcomes being observed particularly in the long-term, the mechanisms underlying individual weight-loss trajectories after bariatric surgery are not fully understood but are likely due to a complex interplay between surgery- and patient-related factors [3].

A patient-related factor for poor weight-loss outcomes frequently reported in the bariatric literature is the presence of problematic eating behaviors, particularly uncontrolled eating (i.e., feeling that one cannot stop eating or control how much one is eating) [4]. The experience of uncontrolled eating has been associated with insufficient weight loss or weight regain by a growing and consistent body of literature [5,6].

In association, negative urgency—the tendency to act impulsively in the face of negative emotions—seems to be in close relation with uncontrolled eating [7,8]. Individuals with higher levels of negative urgency are at greater risk of engaging in more frequent binge-eating [9]. Studies also suggested that elevated levels of negative urgency predict elevated expectations that eating helps to manage a negative affect, which in turn predicts food overconsumption [10]. Finally, eating-related psychopathology—that is, restraint and concerns with eating, shape, and weight—is common in the bariatric surgery population pre- and post-surgery and has been consistently associated with uncontrolled eating [11,12].

Another patient-related factor that requires careful attention among bariatric patients involves the issue of excess hanging skin following the massive weight loss induced by the surgery. Surplus skin is very common (up to 96%) after the bariatric intervention, mainly in the abdominal, upper arm, and inner thigh regions [13]. The associated aesthetics can lead to physical discomfort, significant impairment in health-related quality of life, and psychosocial issues, such as social withdrawal and intimacy problems [14]. This undesirable consequence alters the way patients look at their bodies, becoming extremely self-conscious [15]. Despite the satisfactory weight loss results, continuous feelings of disgust or contempt for body image impacts patients’ psychological wellbeing and leads patients to seek body-contouring procedures [16,17]. For women who submitted to bariatric surgery, the discomfort with the excessive skin showed to be significantly associated with more eating-disorder psychopathology and more depressive symptoms [18].

In close association with the dissatisfaction with the excessive hanging skin, body image shame has also been pointed out as a key risk factor for the development and maintenance of eating-related difficulties [19,20]. Gilbert and Andrews (1998) define shame as an unwanted universal human emotion that emerges in the social context when the self perceives that others or the self evaluate him/her as weak, unattractive, inferior, and/or defective, putting him/her at risk of criticism, judgment, or rejection [21]. Individual responses to feelings of shame are varied. While some individuals can tolerate these types of feelings, others resort to defensive strategies such as hiding, disguising, or camouflaging the parts of the self that are considered sources of shame, which, in turn, tends to precipitate negative emotions (e.g., anxiety, anger, and disgust) [22]. When the focus of shame is weight and body shape, the perceptions of inferiority in comparison to others may predispose people to difficulties in regulating eating behaviour [23,24]. One study revealed a significant and positive relationship between shame and eating-related psychopathology [19]. Consistent with this perspective, another investigation also showed that the relationship between overall shame and eating-related psychopathology is fully dependent on the presence of body image-related shame and consequent attitudes and behaviours [24]. Lastly, past research has identified that greater body image dissatisfaction in women is associated with higher depression [25,26]. Moreover, and as previously suggested, body image shame is extremely frequent in bariatric patients pre- and post-surgery [17,27,28], particularly in individuals with poor psychological functioning [17]. It is not clear whether a direct correlation exists between weight loss induced by bariatric procedures and body image improvement, that is, the rapid change in body shape achieved post-surgery may not be accompanied by psychological changes related to body image. This supports theories about the fragile association between the mental representation of one’s body and one’s real appearance [29]. Thus, considering the aforementioned psychosocial impacts of excessive skin, it is possible that, after bariatric intervention, the discomfort with excessive skin experienced, particularly in patients presenting higher levels of psychological distress, can increase body image shame and, consequently, increase difficulties with the regulation of eating behaviors. 

**Figure 1:**
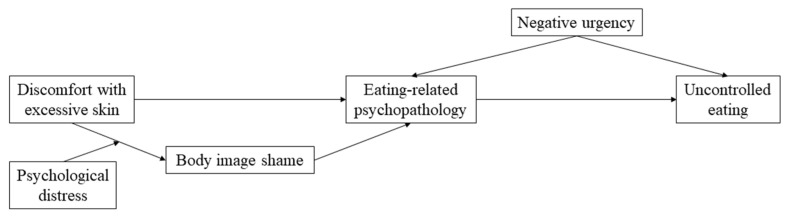
Hypothesized model: Body image shame as a mediator between discomfort with excessive skin and eating-related psychopathology; negative urgency as a mediator between eating-related psychopathology and uncontrolled eating; psychological distress as a moderator between discomfort with excessive skin and body image shame.

Despite the growing body of evidence regarding the association between each of these patients-related factors and disordered eating, to the best of our knowledge, no study has explored how these aspects interact with each other to explain uncontrolled eating, particularly in bariatric surgery populations. Thus, taking into consideration the findings previously described, this study sought to explore the proposed model depicted in Figure 1. In this model, we hypothesize that discomfort with excessive skin, especially in patients with higher levels of psychological distress, would be associated with more body image shame, which would be positively and significantly associated with eating-related psychopathology. In turn, eating-related psychopathology would increase uncontrolled eating via higher levels of negative urgency. Since body image shame in bariatric populations occurs mainly due to excess skin [13,15], understanding how these aspects interact to promote uncontrolled eating behaviors might have important implications for clinical practice.

## 2. Materials and Methods

### 2.1. Procedure and Participants

This cross-sectional study is part of a larger longitudinal study conducted at a central hospital in the north of Portugal that assessed patients undergoing primary and reoperative bariatric surgery preoperatively and at different follow-up times [30]. For this study, patients were included if they were more than 12-months post-surgery. Participants were invited to the study after the medical follow-up appointments at the hospital and responded to a set of self-report measures. This assessment was separate from the treatment, as usual protocol of the multidisciplinary team. All participants were informed about the aims of the study and the voluntary nature of their participation and signed an informed consent form. Participants were aware that the information collected during their participation in this study was not to be shared with the multidisciplinary team.

Specific exclusion criteria included a severe cognitive compromise that limited patients’ autonomy, acute presentation of psychiatric conditions with severe impairment of global functioning, pregnancy during the period until the follow-up assessment, not being able to understand written and spoken Portuguese, and not having been submitted to any body-contouring surgery. 

### 2.2. Measures 

#### Self-Report Measures

Sahlgrenska Excess Skin Questionnaire (SESQ) [31] is a self-report measure used to assess patients’ experience of excess skin after massive weight loss. This measure comprehends, on its original version, 29 items. However, for this study, only 11 items were used: two items about the number of body parts where the subjects consider themselves to have excess skin (“Do you consider that you have excess skin in how many areas of your body? Which are?”); and nine combined items about the impact of excess skin on daily life routines, answered on a four-point Likert scale from 0—“always” to 4—“never”. These nine items were combined to produce a global score where higher scores are indicative of less discomfort with excessive skin. Cronbach’s α for our sample was 0.78.

Body Image Shame Scale (BISS) [24] is a self-report measure to evaluate body image shame in its external and internal dimensions. For the present investigation, only a global score was used, with higher scores indicating greater body image shame. Cronbach’s α for our sample was 0.92 for the total score. 

Depression Anxiety Stress Scale (DASS) [32,33] is a self-report measure used to assess depression, anxiety, and stress symptoms. In this study, only the global score was used, with higher scores expressing greater psychological distress. Cronbach’s α for our sample was 0.96 for the total score. 

Eating Disorder Examination Questionnaire (EDE-Q) [34,35] is a self-report measure used to assess eating-disorder psychopathology and associated features through four subscale scores—restraint, eating concern, shape concern, and weight concern—as well as a global score. For this study, only the global score was used. Higher scores indicate greater eating-disorder psychopathology. Cronbach’s α for our sample was 0.83 for the global score.

Three-Factor Eating Questionnaire—R21 (TFEQ_R21) [36,37] is a measure used to study different types of eating behaviors. Only the uncontrolled eating subscale was used to assess the tendency to overeat as a result of loss of control over eating. Higher scores express more uncontrolled eating. Cronbach’s α for our sample was 0.91 for this subscale.

Urgency, Premeditation, Perseverance, and Sensation-Seeking scales (UPPS-NU) [38] is a measure that assesses the impulsivity based on the Five-Factor Model of personality. For this study, only the negative-urgency subscale was used, with higher scores indicating a greater tendency of the individuals to act impulsively under negative emotions. Cronbach’s α for our sample was 0.88 for the total score.

### 2.3. Statistical Analysis

The different weight-related variables were computed as follows: body mass index (BMI): weight/(height2); percentage of BMI lost (%BMIL) = ((BMI_pre-surgery – BMI_current)/BMI_pre-surgery) × 100; Weight regain (kg) = BMI_current – BMI_lowest. BMI_pre-surgery represents the BMI before the participant was submitted to bariatric surgery; BMI_current represents the BMI at the follow-up assessment; and BMI_lowest represents the lowest BMI achieved since surgery.

The normality of all variables was analysed, and non-parametric tests were used when the assumptions for normality of the data were not assumed. Descriptive statistics were used to examine sociodemographic, weight-related, and psychological characteristics. Partial correlations analyses controlling for follow-up assessment, primary vs. reoperative, and type of surgery were used to investigate the relationship between the variables under study. This set of analyses was performed using the IBM^®^ SPSS^®^ Statistics 24 (IBM Corp, Armonk, NY, USA). 

The path analyses were conducted using the “lavaan” package [39] for the R statistical environment (version 4.0, R Development Core Team, 2019, Vienna, Austria). Model goodness fit was examined using the following indices: chi-square statistic/degrees of freedom (χ2/df) with values smaller than 5; the Tucker–Lewis index (TLI) and the comparative fit index (CFI) with values > 0.95; the root mean square error of approximation index (RMSEA) ≤ 0.06; and the standardized root mean square residual (SRMR) ≤ 0.08 [37]. Variables with *p* values ≤ 0.05 were considered statistically significant [40,41]. 

## 3. Results

The study included 137 women aged between 24 and 67 years (M = 46.41, SD = 10.659) who submitted to primary (*n* = 62, 45.3%) or reoperative (*n* = 75, 54.7%) bariatric surgery. Five participants refused participation in the study, and six were pregnant. Ninety-one (66.4%) participants underwent gastric bypass, and 42(30.7%) received gastric sleeve. Most women were married (*n* = 86, 49.6%), and 23 (16.8%) were single. Thirty-seven (27.0%) participants had completed elementary school (5 years or less), and 25 (18.2%) had completed high school (9–12 years). The majority of women were employed (*n* = 72, 52.6%), while 35 (25.5%) were unemployed. Sixty-one (44.5%), 31 (22.6%), 20 (14.6%), and 25 (18.2%) participants had 12-, 18-, 24-, and 36-months follow-up, respectively. 

Considering the excessive skin, only 25 (18.2%) participants reported not feeling any discomfort with excess skin, 34 (24.8%) participants reported discomfort in only one body area, and 77 (56.2%) participants presented discomfort in two parts of the body. Table 1 presents detailed sociodemographic, weight-related, and psychological information of our sample. 

Correlation analysis between variables under study (Table 2) suggested that discomfort with excess skin (SESQ) was negative and statistically significantly correlated with body image shame (BISS), eating-related psychopathology (EDE-Q), and psychological distress (DASS). In turn, body image shame (BISS) presented positive and statistically significant correlations with eating-related psychopathology (EDE-Q) and psychological distress (DASS). Lastly, uncontrolled eating (TFEQ_R21) showed positive and statistically significant correlations with eating-related psychopathology (EDE-Q) and negative urgency (UPPS-NU). 

**Figure 2:**
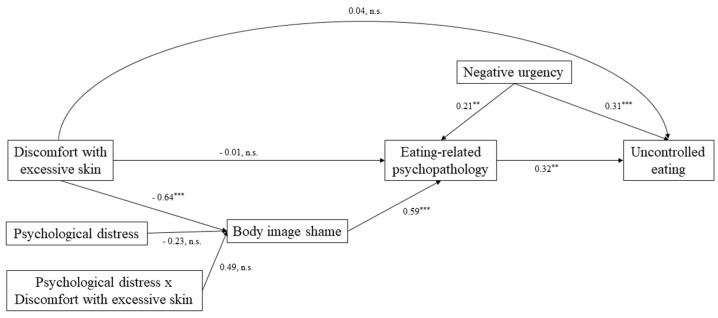
Graphic representation of the mediation model with estimated standardized coefficients: the mediating role of body image shame in the relation between discomfort with excessive skin and eating-related psychopathology and the mediating role of negative urgency in the relation between eating-related psychopathology and uncontrolled eating. SESQ, Sahlgrenska Excess Skin Questionnaire; BISS, Body Image Shame Scale; BISS_EDE_Q, Eating Disorder Examination Questionnaire; DASS, Depression Anxiety Stress Scale; UPPS, Urgency, Premeditation, Perseverance, and Sensation-Seeking scales; TFEQ, Three-Factor Eating Questionnaire; ^**^ *p* < 0.01; ^***^ *p* < 0.001.

Figure 2 depicts the final mediation model and parameters. The final model showed a good fit to our data (χ^2^(6) = 7.561, *p* = 0.272, CFI = 0.990, TLI = 0.976, RMSEA = 0.048, and SRMR = 0.019 (37)). We found that body image shame mediated the relationship between discomfort with excess skin and eating-related psychopathology (Effect = −0.122, SE = 0.060, z-value = −2.025, *p* = 0.04). Additionally, results also suggested that the relationship between eating-related psychopathology and uncontrolled eating was mediated by negative urgency (Effect = 0.067, SE = 0.035, z-value = 1.912, *p* = 0.05). Neither psychological distress and the interaction between psychological distress and discomfort with excessive skin moderated the relationship between discomfort with excessive skin and body image shame (Effect = −0.044, SE = 0.057, z-value = −0.769, *p* = 0.44), (Effect = 0.092, SE = 0.093, z-value = 0.982; *p* = 0.33), respectively.

## 4. Discussion

In this study, we explored how discomfort with excessive skin, body image shame, psychological distress, eating-related psychopathology, and negative urgency interact to understand uncontrolled eating among a sample of women submitted to bariatric surgery.

Overall, our results are in accordance with our hypothesized model and most of the associations between the variables under study. Our data showed that increased discomfort with excessive skin was associated with higher levels of eating-related psychopathology, and this relationship seems to be mediated by body image shame. Contrary to our hypothesis, psychological distress was not identified as a moderator between discomfort with excess hanging skin and body image shame. Interestingly, these data suggest that body image shame was influenced by the discomfort experienced with the surplus skin resulted from bariatric surgery beyond and independently of the individual’s psychological state. These findings are in accordance with previous studies arguing that body image is a multifaceted construct that is not exclusively dependent on weight-loss results [42]. In other words, despite the massive weight loss and effective improvement of co-morbidities after bariatric surgery, the side effect of excess hanging skin can continuously impact individual perception of one’s body, maintaining negative thoughts and behaviours centered around appearance [43,44]. Consequently, negative feelings about body image seem to not serve as a motivator for engaging in more healthy eating thoughts/behaviors but rather increase restraint eating and concerns about food, weight, and shape [19,20]. 

Consistent with past research, our data also suggested an interplay between eating-related psychopathology, negative urgency, and uncontrolled eating. Specifically, the tendency to act rashly under negative emotions seems to be a mediator of the relationship between eating-related psychopathology and uncontrolled eating. That is, the lack of control over impulses is more important than the eating psychopathology to explain the lack of control over eating. These findings are consistent with previous studies, suggesting more support for the argument that impulse control is a central factor in disordered eating behavior [8,12]. 

While this study has significant strengths, several limitations must be acknowledged. The limited number of patients included in our study may prevent us from detecting statistically significant associations. Moreover, some concerns should be made regarding the generality of our findings. Firstly, our sample was constituted exclusively by women, which compromises the generality of our findings. Although research on the field showed that women report more excess-skin discomfort compared to men [43,44], future research should replicate these findings with samples including men. Secondly, the cross-sectional design of our study does not allow us to conclude causality between the variables studied. For example, past research sought to explore preoperative factors that may predict body image concerns after bariatric surgery, demonstrating that postoperative body image concerns were associated with low self-esteem preoperatively [45]. In this way, bariatric surgery candidates presenting psychological risk factors may be more vulnerable to body image shame after bariatric surgery, which can induce disordered eating-behaviors. Longitudinal studies are mandatory to promote a more in-depth understanding of the body image shame construct. Lastly, in this study, we assessed discomfort with excessive skin reported by participants, which allowed us to explore the subjective experience of patients—an aspect often overlooked during treatments. Yet, although understanding patients’ subjective experiences represents important clinical implications, it can, and not rarely, vary from the objective experience. Thus, future investigations exploring differences between self-reported and objective measurements of excess skin would be useful to study whether a distortion of body image is related to the discomfort caused by excessive hanging skin.

The results of our study bring novelty to the field demonstrating that, in women submitted to bariatric surgery, the discomfort with excessive skin in patients with body image shame leads to more eating-related psychopathology which, associated with more negative urgency, prompts uncontrolled eating. The evidence provided in this study strongly supports the clinical impact of excessive skin and body image shame on uncontrolled eating, bringing helpful insight for multidisciplinary teams committed to providing care to bariatric patients struggling with body image and eating difficulties. Future investigations should explore the impact of intervention programs targeting modifiable factors, informing about the potential impacts of excess skin on their daily life, and promoting realistic expectations, body acceptance, and healthy eating behaviors to optimize the results of bariatric surgery. 

## Figures and Tables

**Table 1:** Characterization of the sample: Sociodemographic, weight-related, and psychological information.

	M(SD)/*n* (%)	Min. (Max.)
Sociodemographic and weight-related information		
BMI_pre-surgery	43.74 (5.581)	32.13 (60.96)
BMI_current	30.00 (5.038)	19.84 (46.46)
%BMIL	31.29 (8.742)	−2.08 (52.38)
Weight regain (Kg)	0.76 (1.235)	−0.08 (6.36)
Classification according to BMI (kg/m^2^)	Pre-surgery	Current
Normal (18.5 < IMC < 24.9)	0 (0.0%)	21 (15.3%)
Overweight (25 < IMC < 29.9)	0 (0.0%)	45 (32.8%)
Obesity Class I (30 < IMC < 34.9)	7 (5.1%)	45 (32.8%)
Obesity Class II (35 < IMC < 39.9)	25 (18.2%)	17 (12.4%)
Obesity Class III (IMC > 40)	101 (73.7%)	5 (3.6%)
Psychological information		
SESQ_Global score	28.26 (7.01)	5 (36)
BISS_Global score	16.30 (12.86)	0 (56)
EDE-Q_ Global score	1.52 (1.10)	0 (5.48)
DASS_Global score	13.78 (12.08)	0 (51)
UPPS_Negative urgency	2.10 (0.64)	1 (3.83)
TFEQ_Uncontrolled eating	1.44 (0.51)	0.89 (3.22)

BMI, body mass index; %BMIL, percentage of BMI lost; Weight regain, increased weight (in Kg) after reaching the lowest weight since surgery; SESQ, Sahlgrenska Excess Skin Questionnaire; BISS, Body Image Shame Scale; BISS_EDE_Q, Eating Disorder Examination Questionnaire; DASS, Depression Anxiety Stress Scale; UPPS, Urgency, Premeditation, Perseverance, and Sensation-Seeking scales; TFEQ, Three-Factor Eating Questionnaire.

**Table 2:** Correlations between psychological and weight-related information.

	1	2	3	4	5	6	7	8
1. SESQ_Global score	-							
2. BISS_Global score	−0.565 ***	-						
3. EDE_Q_Global score	−0.426 ***	0.621 ***	-					
4. DASS_Global score	−0.365 ***	0.435 ***	0.370 ***	-				
5. UPPS_Negative urgency	−0.396 ***	0.442 ***	0.453 ***	0.576 ***	-			
6. TFEQ_Uncontrolled eating	−0.074	0.228 **	0.371 ***	0.194	0.393 ***	-		
7. Weight regain	−0.021	−0.020	0.137	0.206 **	0.082	0.145	-	
8. %BMIL	0.169	−0.030	−0.176 **	−0.031	−0.037	−0.150	−0.394 ***	-

SESQ, Sahlgrenska Excess Skin Questionnaire; BISS, Body Image Shame Scale; BISS_EDE_Q, Eating Disorder Examination Questionnaire; DASS, Depression Anxiety Stress Scale; UPPS, Urgency, Premeditation, Perseverance, and Sensation-Seeking scales; TFEQ, Three-Factor Eating Questionnaire; %BMIL, percentage of BMI lost; Weight regain, increased weight (in Kg) after reaching the lowest weight since surgery, ^**^ *p* < 0.01, ^***^ *p* < 0.001.

## Data Availability

The complete dataset, syntax, and output corresponding to the statistical analysis are available upon request.
